# Supplementary material for: Detailed molecular and epigenetic characterization of the pig IPEC-J2 and chicken SL-29 cell lines
Source: iScience. 2023 Feb 20;26(3):106252. doi: 10.1016/j.isci.2023.106252 (PMC10018572; doi:10.1016/j.isci.2023.106252)
Supplement: Data S1. Complete homer output for identified motifs in Pig IPECJ-2, related to Table 2 — Homer motif analysis results for histone modifications H3K4me1, H3K4me3, H3K27ac, and enhancer elements of pig IPECJ2 cell line. P-values >1e-10 are possible false positives. Within each folder (e.g. peak_files_CTCF) are the html files showing the identified motifs when using homer (e.g. homerResults.html). [file mmc2.zip › S5/Pig_IPECJ_2/peak_files_enhancer/homerResults/motif3.similar.html]

motif3

## Information for motif3

C
T
G
A
A
G
C
T
A
G
C
T
G
C
A
T
T
G
A
C
G
T
A
C
C
A
G
T
T
C
G
A
  
Reverse Opposite:  

A
G
C
T
G
T
C
A
A
C
T
G
A
C
T
G
C
G
T
A
T
C
G
A
T
C
G
A
G
A
C
T
  

|  |  |
| --- | --- |
| p-value: | 1e-80 |
| log p-value: | -1.845e+02 |
| Information Content per bp: | 1.626 |
| Number of Target Sequences with motif | 14125.0 |
| Percentage of Target Sequences with motif | 38.55% |
| Number of Background Sequences with motif | 12364.0 |
| Percentage of Background Sequences with motif | 33.80% |
| Average Position of motif in Targets | 150.3 +/- 84.9bp |
| Average Position of motif in Background | 150.7 +/- 90.2bp |
| Strand Bias (log2 ratio + to - strand density) | 0.0 |
| Multiplicity (# of sites on avg that occur together) | 1.27 |
| Motif File: | file (matrix) reverse opposite |

### Similar de novo motifs found

|  |  |  |  |  |  |  |  |
| --- | --- | --- | --- | --- | --- | --- | --- |
| Rank | Match Score | Redundant Motif | P-value | log P-value | % of Targets | % of Background | Motif file |
| 1 | 0.630 | C T A G A G C T C G T A G T C A T G C A G T A C C T G A A G C T | 1e-59 | -136.858710 | 22.44% | 19.03% | motif file (matrix) |
| 2 | 0.772 | C T A G C A T G G T A C T G C A G A T C G A C T A C G T A T G C T G A C G C A T | 1e-53 | -123.946643 | 4.03% | 2.64% | motif file (matrix) |
| 3 | 0.731 | C A T G T A G C A C G T C T G A C T A G A C T G G T C A G C T A G C T A C G T A | 1e-41 | -94.991813 | 18.57% | 15.92% | motif file (matrix) |
| 4 | 0.616 | C A T G A C G T A C G T G A C T A G T C G A T C C A T G T A C G C T G A C T G A | 1e-29 | -67.280539 | 24.86% | 22.36% | motif file (matrix) |
